# The Application of Entrepreneurial Elements in Mathematics Teaching: Challenges for Primary School Mathematics Teachers

**DOI:** 10.3389/fpsyg.2022.753561

**Published:** 2022-03-23

**Authors:** Muhammad Sofwan Mahmud, Siti Mistima Maat, Roslinda Rosli, Nur Ainil Sulaiman, Shahrul Badriyah Mohamed

**Affiliations:** ^1^Faculty of Education, Universiti Kebangsaan Malaysia, Bangi, Malaysia; ^2^State Education Department, Ministry of Education Malaysia, Putrajaya, Malaysia

**Keywords:** entrepreneurial elements, mathematics teaching, application, challenges, primary schools

## Abstract

The entrepreneurial element is one of the aspects emphasized in the primary school mathematics education curriculum in Malaysia. However, previous studies have found that application of entrepreneurial elements in mathematics teaching is still lacking. This study was therefore conducted to identify the real challenges that mathematics teachers face in applying the entrepreneurial element in mathematics teaching. This study is qualitative case study which involved six primary school mathematics teachers. Semi-structured interviews, observation, document analysis and field notes were used for the data collection process in this study. The data obtained was analyzed using the constant comparative analysis method to determine themes and sub-themes. The findings were that a teacher’s knowledge, teaching style, limited time of teaching, and attitudes, and in-service courses and training are among the identified challenges in applying entrepreneurial elements in mathematics teaching. This study expands knowledge and the literature on the challenges faced by school mathematics teachers in applying entrepreneurial elements in mathematics teaching. Various initiatives need to be taken to ensure that all the challenges can be overcome, and ultimately enable the entrepreneurial elements to be applied to students more widely.

## Introduction

Entrepreneurial elements are important and need to be applied to students who are both teaching and learning mathematics (Jamil et al., 2015). This is one of the cross-curricular elements that emphasizes the various skills that need to be mastered by students in addition to various skills and concepts in mathematics (Ministry of Education Malaysia, 2014). The application of entrepreneurial elements in mathematics teaching is part of an important effort to shape the characteristics and practices of entrepreneurship as a culture among students. In general, mathematics learning based on the existing mathematics syllabus already includes basic elements related to entrepreneurship, such as problem-solving related to money calculation; elements of buying and selling; and basic terms such as profit, loss, dividends, and many more that can form the basis of a student’s thinking and behavior related to the field of entrepreneurship (Ministry of Education Malaysia, 2014). There are also many others entrepreneurship elements which are essential to be integrated in teaching activities in order to create an entrepreneurial attitude and culture in the daily lives of students. According to Curriculum Development Division (2011), the application of entrepreneurial elements refers to the application of elements that can lead students to have behaviors, ways of thinking, manage basic projects to plan a simple sales project, produce products based on technological and vocational knowledge and skills, and have considerations that take moral and ethical values into account in daily life. In general, the incorporation of entrepreneurial features is a strategy to intrigue students’ entrepreneurial potential.

In order to develop human capital with entrepreneurial characteristics, students need self-practices that will encourage behavior geared toward personal, family and national well-being. Five elements of entrepreneurship are often integrated in mathematics learning (see Table 1): practicing an entrepreneurial attitude; practicing an entrepreneurial way of thinking when necessary; practicing simple basic sales management methods; producing knowledge-based products, as well as technological and vocational skills; and adopting values, good morals and ethics in the context of entrepreneurship (Curriculum Development Division, 2011). It is not easy to apply all these elements to lessons, but it takes continuous effort and time, so that all the elements are completely embedded in students. These five elements emphasize the knowledge, skills, and values to be taught to students.

**TABLE 1:** Entrepreneurial elements applied in teaching (Curriculum Development Division, 2011).

Entrepreneurial elements	Applied practices
Practising an entrepreneurial attitude	– Being responsible for decisions – Being sensitive to opportunities – Daring to take risks – The power of creativity and innovation – Flexibility – The desire for immediate feedback – Being future-oriented – Willingness to learn from mistakes – Ability to lead – Being achievement-oriented – Being resilient – Tolerating high uncertainty – High endurance – Ability to build social networks
Practicing a way of thinking about entrepreneurship when necessary	– Observe the environment intentionally and purposefully. – Analyze observations critically and creatively. – Generate ideas from observations – Choose the best idea from many ideas. – Improve selected ideas (innovation) – Evaluate ideas critically in the context – Implement ideas in the form of abstract or concrete technological products – Adapt new ideas to the needs of society and the environment – Continue to improve the quality of ideas.
Practicing simple basic sales management methods	– Plan a project carefully. – Implement a project according to the steps provided – Monitor projects – Evaluate project implementation
Producing products based on knowledge, as well as technological and vocational skills	– Produce products based on knowledge as well as technological and vocational skills – Use different technologies to produce the same product – Use a variety of sources/alternative sources/recycling sources
Practicing good moral and ethical values in the context of entrepreneurship	– Principles of social responsibility – The principle of justice – Principles of human rights – The principle of autonomy – The principle of transparency

*Source: Curriculum Development Division (2011), Ministry of Education Malaysia.*

The application of entrepreneurial elements in the teaching of mathematics has many purposes and functions. Ezeudu et al. (2013) asserted that entrepreneurship education gives students the ability to foresee and adapt to changes. In their view, entrepreneurship in mathematics education functions as the basis for:

a. Recognizing students who have entrepreneurial characteristics.
b. Enhancing students’ motivation to set up and run their own small sized business or enterprise.
c. Developing fundamental knowledge and skills to encourage self-employment and alternatives to wage empowerment.

In addition, Bolarinwa (2001) in Ezeudu et al. (2013) identify several potential advantages of entrepreneurship education, which are:

a. It will enable students to improve their basic knowledge in operational and admiration of a company, and familiarize with business environment technology changes micro-enterprises.
b. It will play a facilitating role in the development of professional knowledge, career skills and work experience.
c. It will provide opportunities to gain work experience, and managing their own financial (earn, saving, investment) at early stage, boost their confidence, skills and self-esteem.
d. Decreasing unemployment rate in society by promoting and self-employment and business ownership among graduates.

Teachers are encourage to use various approaches in applying entrepreneurial elements in mathematics teaching and learning activities, including the incorporation approach, integration approach, and application approach (Curriculum Development Division, 2011). In the incorporation approach, teachers are given the freedom to choose the approach they want to use depending on their activities, and their teaching and learning time on the day. The incorporation approach is highly appropriate if the teaching activities chosen on the day are explicitly entrepreneurial (Palmér and Johansson, 2018). Entrepreneurial elements are implemented from the beginning to the end of teaching and learning process in mathematics. In the integration approach, entrepreneurial elements can be applied in the teaching and learning of mathematics if the activities planned for any topic don’t otherwise include discussing matters related to entrepreneurship (Haara and Jenssen, 2016).

Teachers can apply any entrepreneurship element in these activities, by asking for their students’ views on relevant situations. Teachers are free to choose any part of the teaching activities to implement entrepreneurial elements (Deveci and Seikkula-Leino, 2018). In the application approach, teaching and learning mathematics is implemented as planned by the teacher, but once the planned teaching objectives are achieved, the entrepreneurial element is applied by relating the mathematical learning outcomes to any relevant entrepreneurial elements. This can be implemented by relating what was learned that day to an actual entrepreneurial situation (Karimi et al., 2016).

In order to ensure that teachers can successfully apply entrepreneurial elements to students when teaching and learning mathematics, the teachers themselves need to have a strong foundation related to entrepreneurial knowledge (Deveci and Seikkula-Leino, 2018). This is important to help teachers include the various elements of entrepreneurship as a cross-curricular element in their teaching. Although in principle the mathematics education syllabus itself has clear elements related to entrepreneurship, efforts to apply the characteristics of entrepreneurship in learning cannot rely on the syllabus alone. Teachers play an important role in encouraging entrepreneurial spirit. Teachers need to wisely organize and strategise their teaching and learning to apply the characteristics of entrepreneurship (Nasir and Safran, 2014). Teachers also need to be creative in attracting students to entrepreneurship. This can indirectly train students to adopt a way of thinking about entrepreneurship where necessary (Ruskovaara and Pihkala, 2015). Teachers and educators should continuously develop their professional skills and maximize their effort to be updated and relevant with the current and continuous change in education. Teachers must prepare their mind to accept major changes and be exploratory (Mahmud, 2019). The teaching and learning techniques used should be able to stimulate and expand student minds, and attract students toward entrepreneurship.

The Ministry of Education (MOE) Malaysia has identified the skills and values that every student needs to face the twenty-first century, and one of them is entrepreneurship. Entrepreneurship is one of the life and career skills students need to master that requires more than thinking skills and knowledge (Karimi et al., 2016). Pupils develop life and career skills to face the complex life and career environment in an increasingly challenging world. The application of entrepreneurial elements in learning is still at a low level, hence, needs to be improved (Othman et al., 2012). This is because of the numerous obstacles that teachers face when attempting to incorporate the entrepreneurial element into the teaching of mathematics. In this context, challenges allude to obstacles in which impede teachers from incorporating entrepreneurial components into their mathematical instruction (Nasir and Safran, 2014). Shariffudin and Idris (2010) explain that the entrepreneurial element is the most recent generic skill applied in teaching. However, this is not the case in the teaching of mathematics context. Their study found that mathematics teachers were found to mostly focus on understanding the concepts and procedures of mathematical solutions, without associating entrepreneurial elements. This means that the value-added element of entrepreneurship in mathematics which is emphasized in the curriculum and assessment standard documents cannot be achieved.

Issues related to a teacher’s level of knowledge about entrepreneurial elements are discussed by Syukri et al. (2013). Hashim and Mohammad (2007) claimed that not all teachers have knowledge related to entrepreneurial elements and competencies; this lack of knowledge makes it difficult to apply entrepreneurial elements in mathematics teaching. Depending on the content and pedagogical knowledge framework, knowing the content of mathematics teaching is a necessary but not sufficient condition for teaching. Mathematics teachers should also know their students’ needs and be well versed with the curriculum, teaching strategies, and assessment strategies in order to teach effectively and effectively (Mahmud and Yunus, 2018). So far, limited study has been conducted to specifically identify the challenges that primary school mathematics teachers face in applying the entrepreneurial element to mathematics teaching and learning activities. This needs to be noted because entrepreneurial skills are one of the skills emphasized in the Primary School Standard Curriculum as skills and values for the twenty-first century (Ahmad and Sopiah Abdullah, 2020). Studying the challenges faced by mathematics teachers in applying the entrepreneurial element in mathematics teaching can expand the understanding of various educationalists in finding better solutions for applying the entrepreneurial element in mathematics education. Therefore, this study was conducted to identify the challenges faced by mathematics teachers in applying the elements of entrepreneurship in the teaching of mathematics.

## Methodology

Using a qualitative approach, a case study method was chosen to be the research design. This method fits the research questions that need to be answered, as it allows the real picture related to the practice of oral questioning among teachers to be understood in depth. Merriam and Tisdell (2016) state that the case study method can provide a comprehensive, complete, and in-depth understanding of the case studied.

Six primary schools in a district in Negeri Sembilan, Malaysia were selected as study sites to explore and understand the types of feedback given by teachers in the process of oral questioning in teaching mathematics. The location of this study was selected according to Marshall and Rossman (2014), who suggest that the place where a study is conducted should be accessible, with no barriers to conducting the study, have a high chance of collecting in-depth data, allow the freedom to research, and be somewhere it is easy for study participants to participate in research. The selection of schools in this study also considers time, distance, and cost constraints. This study requires the researcher to repeatedly carry out observations and interviews in depth.

Six primary school mathematics teachers from six different schools were selected as study participants using the purposive sampling method. The selection of study participants for this study was based on the researcher’s criteria and characteristics, namely mathematics option teachers, teaching mathematics subjects in primary schools, and the teacher’s willingness to be involved in this study. The researcher also took into account the recommendations of Miller et al. (2012) in the selection of study participants, in that purposeful sampling should be based on the need to address research questions, and the participants’ willingness to cooperate and be interviewed, and ability to provide the necessary information. The research participants ranged in age from 27 to 50 years (see Table 2). Teacher Ana, Teacher Nadia, and Teacher Ada are all in their twenties and have a combined teaching experience of fewer than 4 years. Teacher Raha, Teacher Azah, and Teacher Roza, on the other hand, were all over 40 years old and had over 20 years of teaching experience. Each participant earned a bachelor’s degree in mathematics education. As a result, the participants were deemed to possess the credibility, knowledge, and abilities necessary to teach mathematics.

**TABLE 2:** Educational background and the teaching experience of the research participants.

	Ana	Nadia	Ada	Raha	Azah	Roza
Age	28	27	28	46	50	44
Mathematics teaching experience	4	3	4	20	23	23
Academic qualification	Bachelor’s Degree in Mathematics with Education					

Furthermore, to guarantee that this study satisfies ethical standards, the researcher received consent from the Education Policy Planning and Research Division, Ministry of Malaysia Education (MOE), and then authorization from the Negeri Sembilan Education Department (JPNS). Following that, all letters of approval obtained from the MOE and JPNS were submitted to the headmaster of each school chosen to conduct the study in order to obtain authorization to perform the study at that school. All study participants have been requested to sign a letter of consent to participate in this investigation. This should be considered as an ethical consideration while conducting a study to guarantee that the study participants accept and are willing to participate in the study with an open heart (Bailey, 2014). Additionally, this letter serves as a safeguard for the researcher in the event of legal challenges or complications (Miller et al., 2012).

Researchers used observation methods, interviews, document analysis, and field notes. Using various data collection techniques can help researchers to triangulate data at the data analysis level and strengthen their results (Miles et al., 2014). This study used semi-structured face-to-face interviews (Creswell and Poth, 2017) to guide the researcher and ensure that the interview process aligned with the objectives and research questions. The analysis in this study was conducted using the continuous comparative method, where data from the verbatim interviews was compared between each study participant and analyzed using Atlas. Ti 8 software through open coding, axial coding, and selective coding for themes and subthemes. Creswell (2014) claimed that validity and reliability refer to how a study’s findings can accurately and consistently represent the phenomenon studied. The researcher therefore used several methods to improve the study’s validity and reliability using the triangulation method, member checking, and peer review.

## Findings

The researcher adopted a pseudonym to represent each study participant in order to maintain the individuals’ confidentiality. Each presentation of the study’s findings was accompanied with excerpts from observations of teachers teaching, interviews with teachers, and field notes. Examples of observation labels include (Azah, P3/12452–12723), where “Azah” refers to the study participant’s name, “P3” refers to Teacher Azah’s third observation, and “12452–12723” refers to the observation period (sentence position in the analyzed teaching transcript document). The researcher then applied the label “SRI” or “II” for the interview transcript, where “SRI” refers to the stimulated-recall interview and “II” refers to the initial interview. For instance, the label (Roza, SRI3/4751–5047) refers to “Roza” (the study participant’s name), “SRI3” (the third stimulated-recall interview with Teacher Roza), and “4751–5047” (the sentence position in the interview transcript). Additionally, researchers use the label “NL” for data containing field notes, as in (Ada, NL/17082018), where “Ada” (name of study participant), NL (field note), and “17082018” refer to the date of the field note, which is August 17, 2018.

The study found that knowledge, teaching style, time constraint, attitude and in-service training are among the challenges faced by primary school mathematics teachers in applying entrepreneurial elements in mathematics teaching.

### Teacher Knowledge

Teachers lack of knowledge about the entrepreneurial element is a major challenge in applying the entrepreneurial element in the process of teaching and learning mathematics. Teacher Azah stated that the lack of knowledge related to the basic elements of entrepreneurship made it difficult for her to implement the cross-curricular elements related to entrepreneurship more widely, as in the following passage:

“I do not have a good knowledge of the entrepreneurial element, so I cannot apply the entrepreneurial element well in the teaching of mathematics implemented” (Azah, SRI2/12366–12422).

Teacher Ada also stated that her lack of knowledge about entrepreneurship elements caused her to focus only on understanding important concepts and procedures in mathematics (Ada, SRI3/23771–23991). She also stated that her poor knowledge related to entrepreneurial elements made it difficult for her to connect it to the content of mathematics lessons taught (Ada, SRI3/30112–30315).

Despite having limited knowledge of the elements of entrepreneurship, teachers were found to directly relate their mathematics teaching activities to the basic elements of entrepreneurship, such as matters related to sales, profit and loss calculations, and calculating discounts and dividends (Nadia, P2/21355–22149). These elements are inherent in the mathematics learning syllabus used today. Teacher Nadia explained this in an interview.

“Although I do not have a strong knowledge of the elements of entrepreneurship, I still emphasize the basic knowledge related to entrepreneurship such as buying and selling, calculating profit and loss and calculating discounts and dividends because it is outlined in the mathematics syllabus” (Nadia, SRI3/14567–14772).

Apart from specific knowledge related to entrepreneurial elements, Teacher Nadia also associated the difficulties and challenges of applying entrepreneurial elements in teaching mathematics with entrepreneurial experience. She explains this in the following interview excerpts:

“If a teacher has no knowledge related to entrepreneurship, but has experience as an entrepreneur or has run a business, then it may be easy for them to explain the entrepreneurial element in the mathematics teaching process. But for people like me, having no experience with running a business makes it difficult to explain the entrepreneurial element in the teaching process that I implement” (Nadia, SRI3/25687-25829).

Teacher Nadia thus explained that having experience as an entrepreneur or who having run a business might help a teacher apply the entrepreneurial element to their teaching activities.

You may insert up to five heading levels into your manuscript as can be seen in “Styles” tab of this template. These formatting styles are meant as a guide, as long as the heading levels are clear, Frontiers style will be applied during typesetting.

### Teaching Styles

The analysis also found that teaching was seen to be more focused on remembering various mathematical formulas and important mathematical procedures, causing the application of entrepreneurial elements to be given less attention. This is reflected in the following interview excerpts:

“In the teaching of mathematics, it is very important for students to remember various important mathematical formulas, and students need to know the procedures for mathematical solutions” (Raha, SRI2/21344–21467).

“What is more important is that students can remember the methods to solve various mathematical problems to help them in the exam” (Ana, SR1/22718–22765).

The study participants quoted above focused more on helping students to remember various important mathematical formulas and various problem-solving procedures. The entrepreneurial element was therefore given less attention.

The study also found that a lack of contextual mathematics teaching activities related to entrepreneurship also contributes to the challenges in applying entrepreneurial elements in the mathematics teaching process (Ana, NL/22072018). Teaching activities that are more focused on information and are “chalk and talk” in nature make it difficult for the entrepreneurial element to be applied more effectively (Nadia, NL/17092018). Teacher Azah also explained this, as in the passage below:

“Another thing I think is that the teaching of teachers, especially new teachers or teachers who are practising, is more in the form of giving information. More contextual teaching activities are less implemented, and even students play less of a role. Teachers should be able to use role-play, project-based learning and problem-based learning, and be associated with entrepreneurial elements in their teaching activities. Only then can students understand the elements of entrepreneurship, and at the same time, they can appreciate the elements” (Azah, SRI3/20344–41566).

In the quotation above, Teacher Azah explained that mathematics teaching is more descriptive and involves fewer contextual activities, and this problem is seen more often among new teachers. Teachers also suggested that teaching activities should be diversified by allowing the wider role of students in understanding and appreciating the entrepreneurial elements being applied.

### Time Constraint

The study also found that the time allocated for mathematics teaching was not enough to apply the entrepreneurial element in mathematics teaching activities. Teacher Raha explains this in the following interview excerpt:

“The time available to teach mathematics is very short, and that is why teachers cannot focus on applying the entrepreneurial element in mathematics teaching” (Raha, SRI3/21355–22431).

Teacher Raha also added that the lack of time, coupled with too many topics in the mathematics syllabus, means teachers have to rush to finish the syllabus, causing the entrepreneurial element to be given less attention (Raha, SRI3/22548–22639). The limited time also means mathematics teaching has to focus more on aspects of understanding mathematical concepts, rather than the entrepreneurial element.

### The Attitude of the Teacher

The study also found that teacher’s attitudes are important in applying entrepreneurship in mathematics learning. Teachers argue that the teaching and learning of mathematics should focus more on aspects of understanding mathematical concepts and aspects of high-level thinking skills. Teacher Ada explains this in the following excerpt from her interview:

“In my opinion, it is better to focus on the aspect of understanding mathematical concepts and thinking skills to solve mathematical problems because that aspect is more tested in the exam” (Ada, SR1/24569–24611).

Based on the above quote, Teacher Ada does not seem to see the entrepreneurial element as important in teaching mathematics, and prefers to focus on understanding the concepts and procedures in solving mathematical problems.

The study also found that mathematics teachers are less confident about applying the entrepreneurial element in their mathematics teaching. This is due to a lack of knowledge and skills in pedagogy and lesson content related to entrepreneurship, as explained by teacher Ana in the following interview excerpts:

“For new teachers like me, I still lack the confidence to apply the entrepreneurial element in the teaching of mathematics because of my pedagogical knowledge and skills, as well as the content of the mathematics syllabus, are not yet established” (Ana, SRI4/12667–12733).

### In-Service Courses and Training

The analysis also found that the courses and training provided by the authorities related to the application of entrepreneurial elements in the teaching of mathematics do not focus on the application of entrepreneurial elements. This can be seen in the following interview excerpts:

“I think there is less training and courses for teachers related to the application of entrepreneurial elements in the teaching of mathematics” (Nadia, SRI2/21102–21412).

“… I am less skilled in applying entrepreneurial elements in teaching because less training is provided focusing on the application of entrepreneurial elements in teaching…” (Ada, SRI3/27611–27723).

The above passages explain that the lack of training related to applying entrepreneurial elements in mathematics teaching makes it difficult for teachers to better apply entrepreneurial elements. Teacher Azah further explained that the training provided focused more on teaching mathematics itself and less on the entrepreneurial aspect.

“Another thing is the lack of training that focuses on cross-curricular elements related to entrepreneurship. Most of the training is more focused on improving students’ thinking skills in mathematics” (Azah, SRI4/21233–21336).

“The training is there, but training related to the application of entrepreneurial elements in the teaching of mathematics is not often touched on” (Roza, SRI2/23114–23314).

Teacher Ada also explained that the lack of training and exposure for teachers regarding how to apply entrepreneurial elements meant that teachers lacked knowledge about entrepreneurial elements and that it was difficult for them to apply entrepreneurial elements in their teaching activities (Ada, SRI3/21101–21319).

The representation system attending courses or in-service training results in less accurate knowledge gained during the courses or training provided cannot be disseminated to teachers in the school after returning from the course. Teacher Raha explains this in the passage below:

“Sometimes, teachers who are not in the field represent the school on a course, and the information received from the course cannot be disseminated properly to other teachers in the school” (Raha, SRI3/24522–24678).

According to the study’s findings, mathematics teachers face a number of challenges while integrating the entrepreneurial aspect as one of the cross-curricular elements in mathematics instruction. This issue must be addressed so that all issues that occur can be resolved and the entrepreneurial aspect can be effectively extended to students.

## Discussion

The study found that there are various challenges in applying entrepreneurial elements in the process of teaching mathematics, namely teacher knowledge, time allocated for teaching, teacher attitudes, courses and in-service training and teaching styles. All these challenges need to be faced and resolved to improve the application of entrepreneurial elements in mathematics teaching.

Findings show that the lack of entrepreneurial knowledge among teachers means make the mathematics teaching activities implemented only focus on improving students’ understanding of mathematical concepts, without leaving space for the application of entrepreneurial elements. This indirectly contributes to the unwillingness and lack of confidence in mathematics teachers to apply entrepreneurial elements in mathematics teaching. In addition, a teacher’s strong knowledge of the content of mathematics and pedagogy are strongly correlated in the implementation of effective mathematics teaching (Gess-Newsome, 2015). A good knowledge of pedagogical aspects can also help teachers better apply entrepreneurial elements when teaching mathematics. Borko and Livingston (2008) relate this to pedagogical reasoning. They explain that pedagogical reasoning emphasizes the way teachers can change their content knowledge into a form of pedagogy that is suitable and appropriate for the diversity of student abilities and backgrounds. Chan and Yung (2018) suggest that the knowledge structure possessed by a mathematics teacher could affect a teacher’s pedagogical reasoning in the mathematics teaching process. This knowledge needs to be supported by the strength of a teacher’s knowledge related to the content of mathematics lessons because strong teacher knowledge related to the content of mathematics lessons allows teachers to relate teaching to broader entrepreneurial elements and provide more effective feedback in the teaching of mathematics (Palmér and Johansson, 2018).

Knowledge is the key in ensuring that the entrepreneurial element can be applied effectively in the mathematics teaching process (Nasir and Safran, 2014). A solid knowledge of the elements of entrepreneurship can help mathematics teachers relate the various elements of entrepreneurship as cross-curricular elements in mathematics teaching. In this context, planned teaching activities should be more focused on the broader aspects of entrepreneurial knowledge, and not just related to buying, selling and calculation alone. For example, adopting a way of thinking about entrepreneurship when necessary can help teachers apply various skills such as analyzing observations critically and creatively, generating ideas from observations and improving selected ideas (innovation). This will indirectly boost a teacher’s teaching activities as regards divergent thinking, and encourage students to think at a higher level (Curriculum Development Division, 2011).

The study also found that the teaching time allocated for mathematics was insufficient, and made the entrepreneurial element difficult to apply in mathematics teaching. The relatively dense mathematics syllabus of 18 topics that needs to be completed in a year means that teachers rush to finish the syllabus in a short time (Rashid, 2016). This has caused teachers to focus more on understanding mathematical concepts in their teaching and not applying entrepreneurial elements. Teachers need to plan teaching activities wisely to overcome this problem, such as combining various mathematics learning skills so that the time allocated to implement mathematics teaching can be optimized and, in turn, allow various elements of entrepreneurship to be improved.

The attitudes of the teachers themselves, who may feel that it is not important to apply the entrepreneurial element in mathematics learning also contributes to the challenge of applying the entrepreneurial element in the mathematics teaching process. This is because most teachers think that the teaching and learning of mathematics should focus more on aspects of understanding mathematical concepts and aspects of high-level thinking skills. It also indirectly shows the misunderstanding and misconceptions of teachers about the importance of applying entrepreneurial elements in mathematics teaching. However, this has to do with teachers’ effectiveness in applying entrepreneurial elements in the mathematics teaching process. The study of Huber et al. (2014) explained that low teacher effectiveness could affect efforts in applying entrepreneurial elements in teaching activities. A teacher’s positive attitude and high effectiveness in applying the elements of entrepreneurship can make them more creative and more dynamic when applying the elements of entrepreneurship and teaching mathematics.

The study also found that in-service courses and training do not focus on the application of entrepreneurial elements in the teaching of mathematics. This needs to be noted, because adequate courses and teacher training on applying the entrepreneurial element in the mathematics teaching process allow the entrepreneurial element to be properly applied to students in mathematics teaching. This is especially important for new teachers who are still inexperienced in implementing effective teaching (Wolff et al., 2015). New teachers need more exposure to the application of entrepreneurial elements so that their lesson plans can be connected to the various elements of entrepreneurship. The study also found that although courses and training were provided on how to implement cross-curricular elements in mathematics teaching, the focus on entrepreneurial elements has been given less specific attention. There is thus a need for specific courses and training related to the application of entrepreneurial elements in mathematics teaching so that a teacher’s knowledge and skills involving entrepreneurial elements can be developed (Syukri et al., 2013). Teachers also need to be more proactive in finding and acquiring the various knowledge and skills required to apply the entrepreneurial element in mathematics teaching. This can help teachers increase their knowledge and increase their level of self-direction.

The study also found that the teaching styles of many teachers were also relatively one-way, it which it mostly focused on remembering various mathematical formulas and important mathematical procedures, hence, less attention on the application of entrepreneurial elements. This makes it difficult for teachers to apply entrepreneurial elements in the teaching of mathematics. Teachers should therefore be flexible and dynamic in implementing teaching, so that entrepreneurial elements can be applied more interestingly. Teachers should also not be strictly focused on their lesson plans, and should always be able to modify their teaching, especially to meet the various needs of students. In order to ensure the more effective implementation of mathematics teaching that applies various elements of entrepreneurship, teachers need to ensure that it is contextual, so that the entrepreneurial elements that are applied can be better understood and appreciated by students (Tambychik and Meerah, 2010). A contextual approach will enable students to see and feel for themselves the elements of entrepreneurship that are being applied, and help them better understand. Learning activities such as problem-based learning and project-based learning can also be used by teachers in better applying entrepreneurial elements for students (Fancher and Norfar, 2019). This method exposes students to real experiences, and meets the requirements of “learning by doing.”

## Conclusion

There are various challenges faced when applying the entrepreneurial element in mathematics teaching. Hence, all stakeholders should play their part in overcoming these challenges. This study implies that teachers should not perceive this challenge as a problem, and should be proactive in finding solutions to overcome it. The impact of Movement Control Orders (MCO) due to the spread of the COVID-19 epidemic has increased Malaysia’s unemployment rate and this issue cannot be ignored if we wish to ensure better economic development. The application of entrepreneurial elements in teaching prepares students for entrepreneurial attitudes and entrepreneurial thinking. It also helps the students to practice managing simple basic sales projects, to produce products based on knowledge and technological and vocational skills, and finally, to practise moral and ethical values in the context of entrepreneurship. All these elements should be implemented in accordance to the mastery level of primary school students in various disciplines.

## Data Availability Statement

The original contributions presented in the study are included in the article/supplementary files, further inquiries can be directed to the corresponding author.

## Author Contributions

MM conceived and designed the study. SMM and RR collected and organized the database and performed the analysis. MM, NS, SMM, RR, and SBM co-wrote the manuscript and contributed to manuscript revision. All authors read and approved the final submitted version.

## Conflict of Interest

The authors declare that the research was conducted in the absence of any commercial or financial relationships that could be construed as a potential conflict of interest.

## Publisher’s Note

All claims expressed in this article are solely those of the authors and do not necessarily represent those of their affiliated organizations, or those of the publisher, the editors and the reviewers. Any product that may be evaluated in this article, or claim that may be made by its manufacturer, is not guaranteed or endorsed by the publisher.
